# Effect of *Hochuekkito* on Alveolar Macrophage Inflammatory Responses in Hyperglycemic Mice

**DOI:** 10.1007/s10753-012-9441-x

**Published:** 2012-02-26

**Authors:** Masayuki Nakayama, Yukihiko Sugiyama, Hideaki Yamasawa, Manabu Soda, Naoko Mato, Tatsuya Hosono, Masashi Bando

**Affiliations:** Division of Pulmonary Medicine, Department of Medicine, Jichi Medical University, 3311-1 Yakushiji, Shimotsuke, Tochigi, 329-0498 Japan

**Keywords:** alveolar macrophage, Toll-like receptor, diabetes mellitus, streptozotocin, inflammatory response

## Abstract

Diabetes mellitus reduces immunological activity and increases susceptibility to various infections. *Hochuekkito* (TJ-41) has been reported to improve the weakened physical condition of various chronic diseases. BALB/c mice were divided into three groups; groups A and B were fed a standard diet, and group C, a TJ-41 diet. Two weeks after starting these diets, hyperglycemia was induced in groups B and C by injection with streptozotocin. Two weeks later, bronchoalveolar lavage was performed. Toll-like receptor (TLR) ligands (TLR2: peptidoglycan, PGN; TLR4: lipopolysaccharide, LPS; TLR5: flagellin, FLG) were used to stimulate alveolar macrophages (AMs), and TNF-α production was measured. Under hyperglycemic conditions and PGN or FLG stimulation, TNF-α production from AMs was significantly reduced in group B compared with group A. However, treatment with TJ-41 (group C) significantly improved the impaired production of TNF-α. These results suggest that, under hyperglycemic conditions, TJ-41 can improve the inflammatory responses of AMs with stimulation of TLR ligands.

## INTRODUCTION

It is well known that patients with diabetes mellitus have decreased immunological activity and have an increased susceptibility to various infections, including lower respiratory tract infections. Several previous studies of patients with community-acquired pneumonia have reported that hyperglycemia was associated with an increased risk of pneumonia-related hospitalization [1].

Alveolar macrophages (AMs) are first-line defense cells targeting invading pathogens and therefore play a central role in innate respiratory host defense [2]. Few reports have investigated the effects of hyperglycemic conditions on the function of AMs, such as decreased phagocytosis and bactericidal activity [3], and depressed respiratory burst [4]. Recent studies focusing on macrophage inflammatory responses under hyperglycemic conditions have mainly been performed using peritoneal macrophages [5–7] and bone marrow-derived macrophages [8, 9], while data on AMs are limited. Lipopolysaccharide (LPS)-induced macrophage inflammatory protein-2 gene expression in diabetic mice [10] and production of tumor necrosis factor alpha (TNF-α), interleukin (IL)-12, and nitric oxide (NO) in diabetic rats infected with *Mycobacterium tuberculosis* were reported to be decreased [11].

Toll-like receptors (TLRs) are cellular receptors that recognize molecular signatures of pathogens and initiate an inflammatory signaling cascade that is critical to the innate immune response. In humans, ten TLRs have been identified which recognize pathogen-specific ligands. TLR2, TLR4, and TLR5 play important roles in bacterial infection: TLR4 recognizes LPS, a major cell wall component of Gram-negative bacteria, whereas TLR2 and TLR5 recognize peptidoglycan (PGN), another bacterial wall component, and flagellin (FLG), respectively. All three TLRs are expressed and functionally active on AMs [12, 13]. When stimulated with a ligand, TLRs induce the production of inflammatory cytokines and provoke natural immune responses. Our preliminary data showed that hyperglycemic conditions cause an impaired responsiveness of AMs to selective TLR ligands by inhibiting the production of pro-inflammatory cytokines [14].

*Bu-Zhong-Yi-Qi-Tang* (*Hochuekkito*; TJ-41) is a *kampo* (Japanese and Chinese traditional) herbal medicine and has been used to improve the weakened physical condition of patients with various chronic diseases. TJ-41 was prepared as a spray-dried powder of a hot-water extract obtained from ten medical plants, including *Astragali radix*, *Atractylodis Lanceae rhizoma*, *Ginseng radix*, *Angelicae radix*, *Bupleuri radix*, *Zizyphi fructus*, *Auranti Nobilis pericarpium*, *Glycyrhizae radix*, *Cimicifugae rhizoma*, and *Gingiberis rhizoma* [15]. TJ-41 has been reported to exhibit a pharmacological immunopotentiating activity [15] and enhance the suppressed reactive oxygen-producing activity of neutrophils in diabetic rats [16]. Additionally, treatment of human monocytic cells (THP-1 cell line) with TJ-41 has been reported to cause slightly increased expression of TLR4 [17].

In the present study, we evaluated the immune-activating effects of TJ-41 by studying its effects on inflammatory responses of AMs from hyperglycemic mice.

## MATERIALS AND METHODS

### Reagents

TJ-41 was provided by Tsumura Co. (Tokyo, Japan). Mouse food was produced by CLEA Japan (Tokyo, Japan) and was supplemented with 2 mg/5 g (0.04%) TJ-41. Streptozotocin (STZ), a known diabetogen, was purchased from Sigma-Aldrich (St. Louis, MO). *Escherichia coli* LPS was purchased from Sigma. *Staphylococcus aureus* PGN and *Salmonella typhimurium* FLG were purchased from Invitrogen (San Diego, CA). PE-labeled anti-murine TLR2 antibody (Ab) and TLR4 Ab were purchased from eBioscience (San Diego, CA). PE-labeled anti-murine TLR5 Ab was purchased from Imgenex (San Diego, CA). Culture media and supplements were purchased from Sigma.

### Animals

Specific pathogen-free male Balb/c mice at 6–8 weeks of age were purchased from Japan SLC (Tochigi, Japan). Animals were housed in standard cages with carefully controlled ambient temperature (25°C) and artificial light (12 h of light from 8:00 am to 8:00 pm) and were fed with standard laboratory chow with or without TJ-41 and tap water at the animal facility of Jichi Medical University. All experiments described in this study were approved by the Institutional Animal Care and Use Committee of Jichi Medical University.

### Administration of TJ-41 and Injection of STZ

**Fig. 1:**
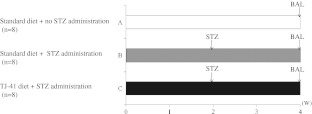
Experimental protocol. Oral administration of TJ-41 or standard diet by gavage for 4 weeks. Two weeks after the beginning of feeding, STZ was injected intraperitoneally to groups B and C. One week after injection, blood glucose levels were measured, and only the mice with blood glucose levels exceeding 200 mg/dl were used in the experiments. Four weeks after the beginning of feeding, mice were sacrificed, before bronchoalveolar lavage (BAL) was performed and blood glucose levels measured.

The experimental setup of this study is outlined in Fig. 1. TJ-41 was administered orally with a composite of 2 mg/5 g (0.04%) per day. Mice were divided into three groups: groups A and B were given standard food, and group C was given food containing TJ-41.

Two weeks after the initiation of TJ-41 treatment, STZ, in 0.01 M citrate buffer (pH 4.5), was injected intraperitoneally at a dose of 250 μg/g body weight into groups B and C. Two weeks later (4 weeks after the beginning of TJ-41 treatment), blood glucose levels were measured using Glutest Ace (Sanwa Chemical Co., Nagoya, Japan) and Glutest sensor (Sanwa Chemical Co.). Only mice with a fasting blood glucose level of at least 200 mg/dl were considered diabetic and used in the following experiments.

### AM Isolation and Culture

Two weeks after STZ injection, tracheas were cannulated with an 18-gauge catheter, and bronchoalveolar lavage (BAL) was performed under deep anesthesia with pentobarbital, by instilling 1 ml of phosphate-buffered saline (PBS) into both lungs through the trachea four times [18]. After each instilment, fluid was collected and pooled. Cells were counted by trypan blue dye exclusion. Cytospins for differential cell counts were prepared and stained with modified Wright-Giemsa (Diff-Quick; International Reagents Co., Kobe, Japan). Cells were resuspended in RPMI 1640 medium supplemented with 10% heat-inactivated fetal calf serum (FCS, Harlan, Indianapolis, IN), 100 U/ml penicillin, 100 μg/ml streptomycin, and 2 mM l-glutamine. Based on trypan blue dye exclusion and differential cell counts, equal numbers of AMs were plated at the indicated cell density. After 2 h of incubation at 37°C in a 5% CO_2_ incubator, nonadherent cells were removed by washing twice with PBS. Adherent cells were cultured in RPMI 1640 medium supplemented with 10% FCS, 100 U/ml penicillin, 100 μg/ml streptomycin, and 2 mM l-glutamine and were either left unstimulated or were stimulated with PGN (10 μg/ml), LPS (100 ng/ml), or FLG (1 μg/ml).

### TNF-α Measurement

AMs plated in 96-well plates at 5 × 10^4^ cells per well were cultured in the medium alone or stimulated with specified reagents at indicated concentrations for 18 h. Cell supernatants were harvested, and levels of TNF-α were measured using commercially available enzyme-linked immunosorbent assay (ELISA) kits (Invitrogen, Carlsbad, CA) according to the manufacturer’s instructions.

### Analysis of Cell-Surface TLR Expression

AMs from control and diabetic mice were obtained by BAL as described above. AMs were resuspended at 1 × 10^5^ cells per 100 μl in staining buffer (PBS containing 1% bovine serum albumin and 0.1% sodium azide). Nonspecific staining was blocked by incubation with anti-murine CD16/32 Ab (eBioscience) for 15 min. After blocking, cells were incubated for 30 min with PE-labeled anti-murine TLR2, TLR4, and TLR5 Abs. AMs were washed twice with staining buffer and fixed with staining buffer containing 1% formalin. Flow cytometry was performed using a FACS LSR flow cytometer (BD Bioscience, San Jose, CA). Using forward scatter and side scatter parameters to define macrophage populations, 1 × 10^4^ events were acquired. Flow cytometry data were analyzed using the CellQuest Pro software.

### Real-Time PCR

AMs were plated in 12-well plates at 4 × 10^5^ cells per well. Cells were cultured in the medium alone or stimulated with specified reagents, and RNA was isolated at the indicated time points using the RNAqueous Kit (Ambion, Austin, TX) according to the manufacturer’s instructions. Reverse transcription was performed on similar amounts of RNA per group using a High Capacity cDNA Reverse Transcription Kit (Applied Biosystems, Foster City, CA). Specific primers for murine TNF-α, TLR2, 4, and 5 were designed, and PCR was performed in triplicate with Fast Universal PCR Master Mix in the ABI 7500 Fast Real-Time PCR System (Applied Biosystems).

### Statistical Analysis

All data are shown as mean ± SEM of each group. The statistical significance of any difference in each parameter among the groups was evaluated by a Student’s *t* test (Statview, SAS Institute, Cary, NC). A value of *p* < 0.05 was considered statistically significant.

## RESULTS

**Fig. 2:**
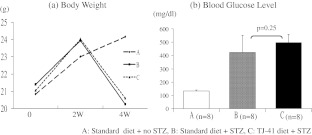
Change in body weight and blood glucose levels in mice after STZ injection. **a** Mouse body weight was measured at 0, 2, and 4 weeks. Body weight of groups B and C (STZ injected) were significantly decreased compared with group A (no STZ). **b** Blood glucose levels of mice after 4 weeks. Data are expressed as mean ± SEM.

To test the immune-activating potential of TJ-41 in the context of diabetic conditions, the drug was administered to mice under both basal conditions and in conjunction with the diabetogen STZ. As shown in Fig. 2, significant body weight loss in animals was observed after 2 weeks in groups B and C, to which STZ was administered. Furthermore, blood glucose levels at week 4 of the study were significantly higher in groups B and C, compared with the untreated control group, but did not differ between groups B and C.

**Fig. 3:**
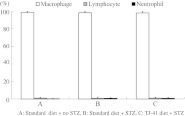
Cell differentiation in bronchoalveolar lavage fluid. There was no difference in cell differentiation in bronchoalveolar lavage between the three groups.

No difference was observed in the cell fractions in the bronchoalveolar lavage fluid among the three groups, and they were mostly macrophages (Fig. 3).

**Fig. 4:**
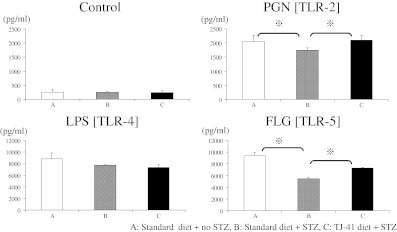
Effect of TJ-41 on TNF-α production in pulmonary-alveolar macrophages. After stimulating AMs with the TLR ligands, PGN (10 μg/ml), LPS (100 ng/ml), or FLG (1 μg/ml) for 18 h, supernatants were harvested, and TNF-α levels were measured in cell supernatants by ELISA. Data are expressed as mean ± SEM.

We initially tested TNF-α production under PGN or FLG stimulation in the three treatment groups. As shown in Fig. 4, production of TNF-α was lower in diabetic (STZ-treated) animals, compared with untreated animals. Interestingly, treatment with TJ-41 significantly increased TNF-α production following PGN and FLG stimulation in diabetic animals (PGN, group B 1,742 ± 80 pg/ml versus group C 2,091 ± 166 pg/ml, and FLG, group B 5,626 ± 288 pg/ml versus group C 7,143 ± 315 pg/ml, *p* < 0.05, respectively) but not following LPS stimulation (Fig. 4). These results suggested that suppressed TNF-α production under diabetic conditions with PGN or FLG stimulation improved with intake of TJ-41.

**Fig. 5:**
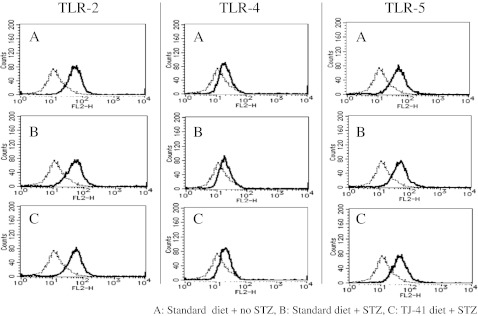
Effect of TJ-41 on TLR surface expression. Flow cytometric analysis of AM surface phenotype was performed by direct immunofluorescence using a BD LSR cell analyzer. AMs were resuspended in staining buffer (1 × 10^5^ cells/100 μl) and nonspecific Fc receptor staining blocked using Fc block. PE-labeled TLR2, 4, and 5 Ab were added to stain the cells before analysis using a FACScan flow cytometer.

We next examined whether the above-described modulation of cytokine production by TJ-41 treatment (Fig. 4) was associated with changes in TLR expression. The expression of TLR2, TLR4, or TLR5 on the cell surface was evaluated by flow cytometry. As shown in Fig. 5, no difference in TLR expression was observed between the three groups. In addition to protein levels, we also tested whether TLR mRNA levels were altered in response to TJ-41 treatment. RT-PCR using specific primers for TLR2, 4, and 5 was performed on cDNA generated from AMs, but no differences were observed in the three treatment groups (data not shown).

Initially, we divided mice into four groups: groups A–C as previously described and one further group which was given food containing TJ-41 but was not injected with STZ. However, in this group, we found entirely no difference comparing with the data of control group A (standard diet + no STZ administration) (data not shown).

To summarize the above results, in diabetic mice, TNF-α production from AMs was significantly reduced following stimulation with TLR2 or TLR5 ligands, compared with the control group. However, TNF-α production in diabetic mice was increased upon treatment with TJ-41. Interestingly, no difference was noted in the expression of TLR mRNA in macrophages or TLR expression on the cell surface between the three groups.

## DISCUSSION

Japanese herbal medicine is a mixture of many plant materials, and the mechanism of action mediating the effect can appear to be complicated. There have been numerous reports describing the beneficial effects of *Bu-Zhong-Yi-Qi-Tang* (*Hochuekkito;* TJ-41) on immune functions. Harada *et al.* reported an enhancement of immune function, particularly of antitumor immunity, through the augmentation of cytostatic activity [19]. Elderly people who received doses of TJ-41 had a significant increase in their serum interferon-gamma (IFN-γ) levels, which is thought to be associated with increased NK cell activity [20]. TJ-41 combined with IFN-γ moderately enhanced the daily activity of chronic fatigue syndrome mice by increasing NK cell activity [21]. Utsuyama *et al.* showed that TJ-41 could enhance the impaired immune function of old mice by increasing the numbers of T and NK cells [22]. Mice administered with TJ-41 for 32 weeks had a significant increase in their splenic NK cell population, and the CD4/CD8 ratio in the spleen was increased [23]. TJ-41 increased IL-18-induced ICAM-1 and CD86 expression, resulting in enhanced TNF-α and IFN-γ production. This suggests that TJ-41 enhances IL-18-induced cell-mediated immunity and may enhance host defense mechanisms against pathogens [24]. Therefore TJ-41 may have beneficial effects via its ability to enhance immune system activation.

As for innate immunity in the diabetic mouse model, most of the studies regarding the impacts of a hyperglycemic state on tissue macrophage inflammatory responses to TLR ligands have been performed on cells other than AMs. Moreover, the examinations have focused on the response to LPS, a TLR4 ligand, but not other TLR ligands.

For example, several studies have shown impaired inflammatory responses in peritoneal macrophages, such as reduced LPS-induced TNF-α and IL-6 production in type 1 diabetic rat models [5, 6] and reduced TNF-α and IL-1β production by LPS plus IFN-γ stimulation in type 2 diabetic (db/db) mice [25]. Contrary to these reports, peritoneal macrophages from diabetic mice produced more IL-1β in response to LPS, resulting in increased peritoneal levels of IL-1β induced by LPS [26]. Also, exposure of bone marrow-derived macrophages derived from non-obese diabetic mice to a hyperglycemic environment resulted in elevated levels of TLR2, TLR4, and TNF-α [9]. Thus, immunomodulatory effects of a hyperglycemic state on macrophage cytokine reactions are complex. Although these data were different in part from our present study, the discrepancy was probably due to the different experimental models, origin of macrophages, and additional stimuli.

Our preliminary data suggested that the hyperglycemic state impairs the reactivity of alveolar macrophages to selective TLR ligands, TLR2 ligand (PGN), and TLR5 ligand (FLG), by inhibiting the production of TNF-α [14]. Additionally, in the present study, the suppressed TNF-α production from alveolar macrophages in mice stimulated with PGN or FLG was alleviated by TJ-41. TNF-α activates T cells, B cells, and macrophages [27]. TNF-α induces the synthesis and release of immunostimulatory polypeptides such as IL-1 and IL-6 from various cells. Together with IL-1, TNF-α acts on a variety of cells like T cells, B cells, fibroblasts, and macrophages to secrete various cytokines which are essential for the development of an effective immune response. These TLRs are involved in responses to Gram-positive bacteria, mycoplasma, and bacteria with flagellar filaments. In contrast, no effect was observed on the level of cell surface receptor expression in this study. Therefore, TJ-41 is likely to have an effect on as yet unidentified intracellular signal pathways.

Diabetes may predispose to increased morbidity and mortality of certain pulmonary infections, such as those caused by *S. aureus*, *Streptococcus pneumoniae*, *M. tuberculosis*, and *Legionella pneumophila* [28]. TLR2 or TLR5 is indeed important in the recognition of these microorganisms. Moreover, recent clinical studies have shown the association of TLR2 and TLR5 gene polymorphisms with susceptibility to infection with *M. tuberculosis* [29] and *L. pneumophila* [30], respectively. In the present study, TNF-α production from alveolar macrophages stimulated with TLR2 or TLR5 ligands during diabetic conditions was decreased, suggesting that inflammatory responses to certain bacteria may be impaired in diabetic patients. Alternatively, TJ-41 recovered suppressed TNF-α production from alveolar macrophages in a hyperglycemic state. This suggests that this drug normalizes the dysregulated inflammatory response in hyperglycemic states and, furthermore, is useful for host biological responses to bacterial infections.

There are several reports which have investigated TLR signaling in hyperglycemic conditions. LPS-dependent TNF-α production in mice increased due to an augmentation of LPS-induced p38 MAPK activity. The extracellular signal-regulated kinase (ERK) MAPK pathway is thought to be critical in TLR3 and TLR7 activation signaling in non-obese diabetic mice [31]. However, several reports have addressed the effects of TJ-41 on TLR signaling. Chino *et al.* reported the effect of *Shi-Quan-Da-Bu-Tang* (*Juzentaihoto*; TJ-48) on TLR4-mediated cellular responses in peritoneal exudate macrophages [32]. Although FACS analysis revealed that TJ-48 had no effect on TLR4 surface expression, it activated the NF-κB and p38 pathways while inhibiting the JNK and ERK pathways. In contrast, Mita *et al.* suggested that TJ-48 increased the expression of TLR4 and might enhance defense against Gram-negative bacteria *in vitro* [17]. Despite the contrasting data in these two reports, the present study and the work by Chino *et al*. suggest that TJ-41/TJ-48 specifically influences the downstream signaling pathways of TLR without affecting its surface expression [32]. Furthermore, the results presented here suggest that TJ-41 modulates MAPK signaling pathways to enhance TNF-α production.

Our study had limitations. We examined only one inflammatory cytokine, TNF-α. There are many inflammatory cytokines involving the site of inflammation. To clarify the exact mechanism of *Hochuekkito*, more examinations will be needed. Furthermore, the differences of the responses of TLR-2 and 4 to hyperglycemic environment between alveolar and other macrophages should be clarified.

## CONCLUSION

Our results suggest that TJ-41 alleviates the suppressed inflammatory responses elicited from AMs following stimulation with TLR ligands in a hyperglycemic state, but the mechanism of this effect requires further investigation.
